# Novel tetrahydronaphthalen-1-yl-phenethyl ureas: synthesis and dual antibacterial-anticancer activities

**DOI:** 10.1080/14756366.2023.2286925

**Published:** 2023-12-07

**Authors:** Yusuf Akbaba, Fatma Necmiye Kacı, Mehmet Enes Arslan, Süleyman Göksü, Adil Mardinoğlu, Hasan Türkez

**Affiliations:** a Department of Basic Sciences, Faculty of Science, Erzurum Technical University, Erzurum, Turkey; b Department of Molecular Biology & Genetics, Faculty of Science, Erzurum Technical University, Erzurum, Turkey; c Faculty of Medicine and Health, St. James’ University Hospital, University of Leeds, Leeds, UK; d Department of Molecular Biology and Genetics, Faculty of Science, Erzurum Technical University, Erzurum, Turkey; e Department of Chemistry, Faculty of Science, Atatürk University, Erzurum, Turkey; f Science for Life Laboratory, KTH-Royal Institute of Technology, Stockholm, Sweden; g Centre for Host-Microbiome Interactions, Faculty of Dentistry, Oral & Craniofacial Sciences, King’s College London, London, UK; h Department of Medical Biology, Faculty of Medicine, Atatürk University, Erzurum, Turkey

**Keywords:** Antibacterial activity, anticancer activity, isocyanates, N,N′-dialkyl urea, phenethylamine

## Abstract

Cancer and antibiotic-resistant bacterial infections are significant global health challenges. The resistance developed in cancer treatments intensifies therapeutic difficulties. In addressing these challenges, this study synthesised a series of N,N′-dialkyl urea derivatives containing methoxy substituents on phenethylamines. Using isocyanate for the efficient synthesis yielded target products **14–18** in 73–76% returns. Subsequently, their antibacterial and anticancer potentials were assessed. Cytotoxicity tests on cancer cell lines, bacterial strains, and a healthy fibroblast line revealed promising outcomes. All derivatives demonstrated robust antibacterial activity, with MIC values ranging from 0.97 to 15.82 µM. Notably, compounds **14** and **16** were particularly effective against the HeLa cell line, while compounds **14**, **15**, and **17** showed significant activity against the SH-SY5Y cell line. Importantly, these compounds had reduced toxicity to healthy fibroblast cells than to cancer cells, suggesting their potential as dual-functioning agents targeting both cancer and bacterial infections.

## Graphical Abstract

**Figure:**
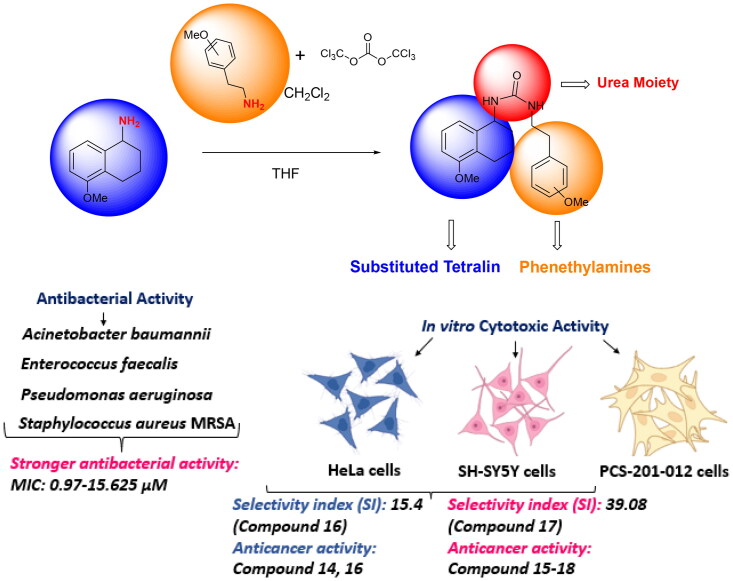

## Introduction

**Figure 1.**
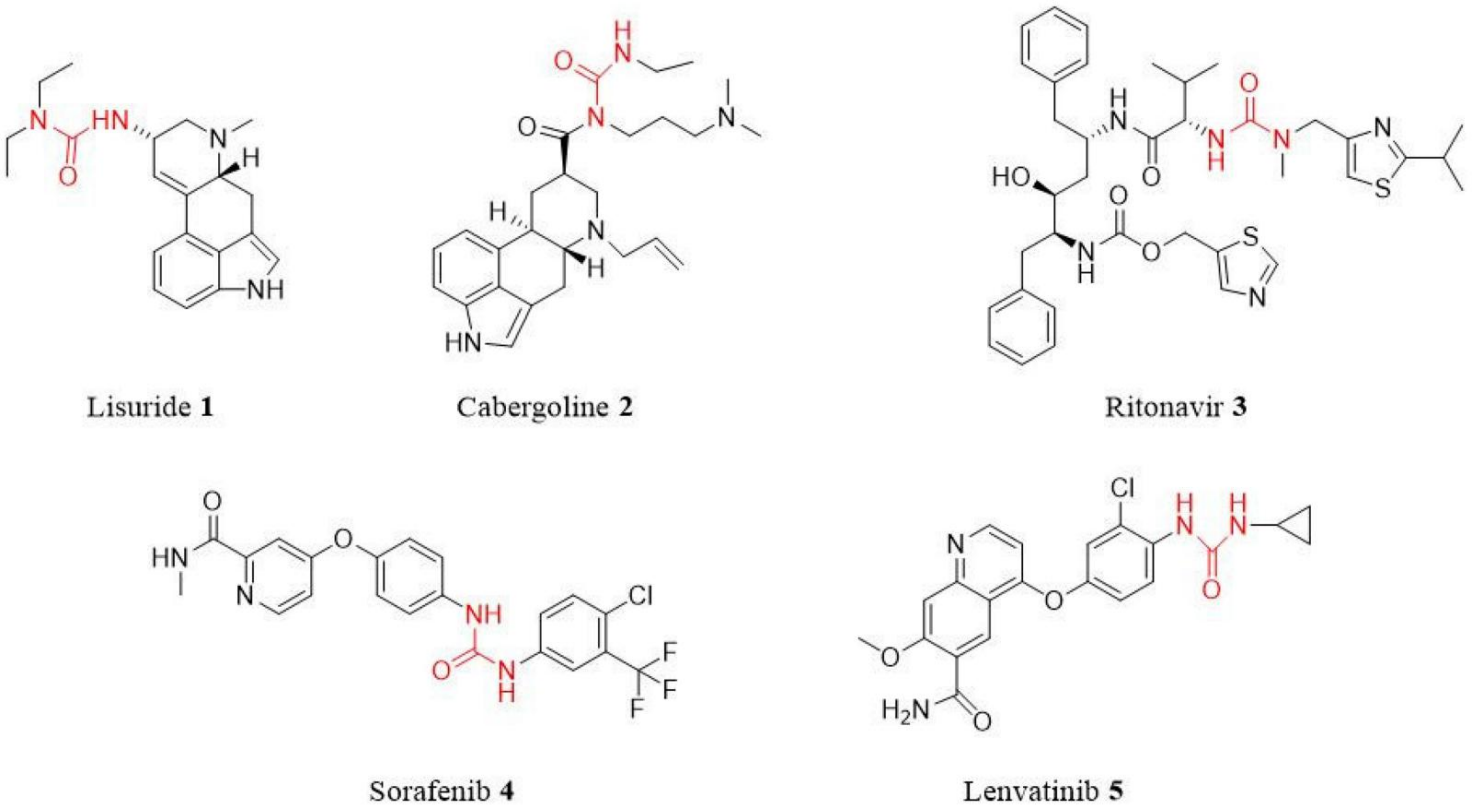
Examples of commercially available urea drugs **1–5**.

Urea derivatives are a large class of biologically active compounds, and molecules with the urea functional group are increasingly used in medicinal chemistry and drug development. Many studies have demonstrated that urea derivatives have a wide variety of biological activities, including antimalarial^1^, antiviral^2^, anti-cancer^3–5^, anti-tubercular^5^, and anti-microbial^6^^,^^7^. Also, extensive biological activity studies on urea compounds have led to the development of some urea-derived drugs. For instance, the urea medication lisuride **(1)**, also known as dopargine, is a serotonin 5-HT2B receptor antagonist^7^. Cabergoline **(2)** is used for the treatment of hyperprolactinaemia and Parkinson’s disease^8^^,^^9^. Ritonavir **(3)** (trade name Norvir) is an antiretroviral drug used as an HIV protease inhibitor^9^. Sorafenib **(4)** (trade name Nexavar) is a kinase inhibitor drug used to treat thyroid, kidney, and advanced primary liver cancer^10^. The anti-cancer medication lenvatinib **(5)** (brand name Lenvima) is used to treat thyroid cancer (Figure 1)^11^.

On the other hand, 2-phenylethylamines and 1-aminotetralins represent crucial building blocks for synthetic organic chemistry and medicinal organic compounds.

**Figure 2.**
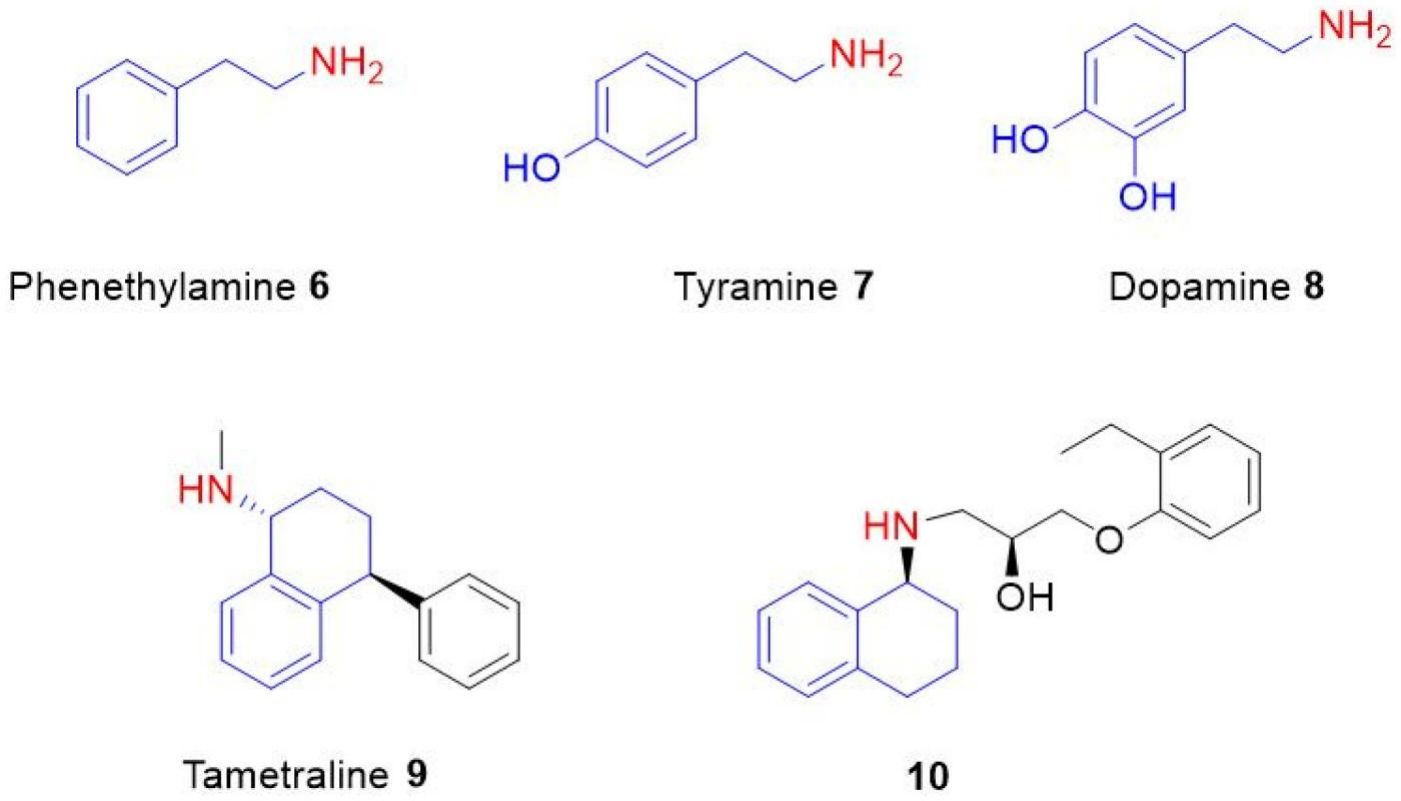
Phenethylamine **6**, tyramine **7**, dopamine **8**, tametraline **9**, and 1-aminotetralin derivative **10**.

For example, phenylethylamine (**6**) is an endogenous trace amine that acts as neuromodulation in the central nervous system of mammals^12^. Tyramine (**7**) is a biogenic amine found in nature and formed from tyrosine by enzymatic decarboxylation^13^. Dopamine (**8**), a hormone-like compound, has a very important role as a neurotransmitter in the brain and body^14^^,^^15^. Tametraline (**9**) is a norepinephrine-dopamine reuptake inhibitor^16^. Aminotetralin **10** is used as a common β3-adrenoceptor antagonist (Figure 2)^15^.

In a previous study, we synthesised ureas with a skeletal structure consisting of 6- and 7-methoxy-1-aminotetralins and methoxy-substituted phenethylamines and investigated their antibacterial, anticancer, and toxicity properties. We have seen that some compounds can be used as anticancer and antibacterial agents^17^.

As can be seen from the information given above, ureas, phenethylamines, and 1-aminotetralins are important biologically active compounds. The presence of these three biologically active groups in one molecule and the high antibacterial and anticancer activity of similar molecules in our previous study led us to this study. Therefore, as an experience from our previous study, we aimed to synthesise a series of N,N-dialkyl ureas containing the urea functional group, 5-methoxy-1-aminotetralin, and substituted phenethylamines in their skeletal structures to investigate their biological properties, such as antibacterial and anticancer activities.

## Materials and methods

### The synthesis of 5-methoxy-1,2,3,4-tetrahydronaphthalen-1-amine (13)

5-Methoxy-1-aminotetralin **13**^18^ was synthesised as described previously.

### General procedure for the synthesis of N,N′-dialkyl ureas (14–18)

A solution of triphosgene (1 mmol) in CH_2_Cl_2_ (15 ml) was cooled to 0 °C. Then, 2-phenethylamine **11**(**a–e**) (3 mmol) dissolved in CH_2_Cl_2_ (5 ml) was added to it in a drop-wise manner. Following this, a NaOH solution (3 mmol) in 7 ml of water was introduced. The mixture was stirred at ambient temperature for 1 h post-addition. The aqueous layer was then separated. The remaining organic layer was rinsed with water three times, each with 20 ml, and dried using magnesium sulphate. Evaporating the filtrate yielded the corresponding isocyanate, which was used as-is without any additional purification. This isocyanate **12**(**a–e**) (1 mmol) was either dissolved or suspended in dry THF (10 ml) and cooled using an ice bath. Separately, 1-aminotetralin **13** (1 mmol) was dissolved in dry THF (5 ml) and then gradually added to the earlier mixture. The combined mixture was stirred at room temperature for 6 h. After this, the solvent was removed by evaporation. The final products, N,N′-dialkyl ureas **14–18**, were obtained through recrystallisation using a solvent mix of EtOAc/hexane at a ratio of 3:1.

#### 1-(5-methoxy-1,2,3,4-tetrahydronaphthalen-1-yl)-3-phenethylurea (14)

The product **14** was obtained as a white powder, yield: 73%, mp 141–143 °C. IR spectrum, *ѵ*, cm^−1^: 3319, 2937, 2368, 1616, 1558, 1467, 1249, 1016, and 762 cm^–1^; ^1^H NMR (400 MHz, CDCl_3_) *δ* 7.33–7.14 (m, 5H, ArH), 7.12 (t, *J* = 7.8 Hz, 1H, ArH), 6.92 (d, *J* = 7.8 Hz, 1H), 6.69 (d, *J* = 8.0 Hz, 1H, ArH), 4.91–4.83 (m, 1H, CH-N), 4.62 (br.d, *J* = 8.4 Hz, 1H, NH), 4.45 (t, *J* = 5.4 Hz, 1H, NH), 3.79 (s, 3H, OCH_3_), 3.41 (dd, *J* = 12.9, 6.6 Hz, 2H, CH_2_), 2.79 (t, *J* = 6.9 Hz, 2H, CH_2_), 2.69–2.50 (m, 2H, CH_2_), 1.96–1.84 (m, 1H, CH_2_), 1.83–1.66 (m, 3H, CH_2_). ^13^C NMR (101 MHz, CDCl_3_) *δ* 157.63 (CO), 156.94 (C), 139.22 (C), 138.93 (C), 128.84 (2CH), 128.56 (2CH), 126.50 (C), 126.37 (CH), 126.35 (CH), 120.74 (CH), 108.00 (CH), 55.30 (OCH_3_), 48.25 (CH), 41.70 (CH_2_), 36.41 (CH_2_), 30.26 (CH_2_), 22.96 (CH_2_), 19.29 (CH_2_). Analysis calculated for C_20_H_24_N_2_O_2_: C 74.05; H 7.46; N 8.63. Found, %: C 74.15; H 7.49; N 8.59. *M*: 336.39.

#### 1-(5-Methoxy-1,2,3,4-tetrahydronaphthalen-1-yl)-3-(2-methoxyphenethyl)urea (15)

The product **15** was obtained as a white powder, yield: 76%, mp 174–176 °C. IR spectrum, *ѵ*, cm^−1^: 3331, 3287, 2928, 2853, 1614, 1574, 1468, 1263, 1242, 1084, 1035, and 745 cm^–1^; ^1^H NMR (400 MHz, CDCl_3_) *δ* 7.22–7.18 (m, 1H, ArH), 7.15–7.11 (m, 2H, ArH), 6.96 (d, *J* = 7.8 Hz, 1H, ArH), 6.88 (t, *J* = 7.3 Hz, 1H, ArH), 6.82 (d, *J* = 8.2 Hz, 1H, ArH), 6.71 (d, *J* = 8.0 Hz, 1H, ArH), 4.99–4.90 (m, 1H, CH-N), 4.68 (br.s, 1H, NH), 4.52 (br.s, 1H, NH), 3.81 (s, 3H, OCH_3_), 3.66 (s, 3H, OCH_3_), 3.35 (t, *J* = 6.6 Hz, 2H), 2.87–2.80 (m, 2H), 2.75–2.50 (m, 2H), 1.98–1.92 (m, 1H), and 1.82–1.74 (m, 3H).^13^C NMR (101 MHz, CDCl_3_) *δ* 157.69 (CO), 157.49 (C), 156.99 (C), 138.93 (C), 130.65 (CH), 127.84 (CH), 127.27 (C), 126.64 (C), 126.40 (CH), 120.87 (CH), 120.67 (CH), 110.36 (CH), 108.04 (CH), 55.33 (OCH_3_), 55.10 (OCH_3_), 48.33 (CH), 40.82 (CH_2_), 31.27 (CH_2_), 30.31 (CH_2_), 23.02 (CH_2_), and 19.27 (CH_2_). Analysis calculated for C_21_H_26_N_2_O_3_: C 71.16; H 7,39; N 7.90. Found, %: C 71.28; H 7.42; N 7.92. *M*: 354.45.

#### 1-(5-Methoxy-1,2,3,4-tetrahydronaphthalen-1-yl)-3-(3-methoxyphenethyl)urea (16)

The product **16** was obtained as a white powder, yield: 74%, mp 155–157 °C. IR spectrum, *ѵ*, cm^−1^: 3308, 2938, 2857, 1616, 1578, 1512, 1468, 1260, 1163, 1045, 849, and 779 cm^–1^; ^1^H NMR (400 MHz, CDCl_3_) *δ* 7.21 (t, *J* = 7.7 Hz, 1H, ArH), 7.14 (t, *J* = 8.0 Hz, 1H, ArH), 6.94 (d, *J* = 7.9 Hz, 1H, ArH), 6.82–6.68 (m, 4H, ArH), 4.98–4.88 (m, 1H, CH-N), 4.42 (br.s, 2H, NH), 3.81 (s, 3H, OCH_3_), 3.79 (s, 3H, OCH_3_), 3.46 (t, *J* = 6.8 Hz, 2H, CH_2_), 2.81 (t, *J* = 6.8 Hz, 2H, CH_2_), 2.70–2.52 (m, 2H, CH_2_), 2.01–1.89 (m, 1H, CH_2_), and 1.78 (d, *J* = 5.8 Hz, 3H, CH_2_). ^13^C NMR (101 MHz, CDCl_3_) *δ* 159.76 (C), 157.67 (CO), 156.93 (C), 140.85 (C), 138.93 (C), 129.50 (CH), 126.47 (C), 126.31 (CH), 121.13 (CH), 120.72 (CH), 114.45 (CH), 111.79 (CH), 108.01 (CH), 55.26 (OCH_3_), 55.12 (OCH_3_), 48.23 (CH), 41.56 (CH_2_), 36.45 (CH_2_), 30.27 (CH_2_), 22.94 (CH_2_), and 19.27 (CH_2_). Analysis calculated for C_21_H_26_N_2_O_3_: C 71.16; H 7.39; N 7.90. Found, %: C 71.22; H 7.41; N 7.88. *M*: 354.45.

#### 1-(3,4-Dimethoxyphenethyl)-3-(5-methoxy-1,2,3,4-tetrahydronaphthalen-1-yl)urea (17)

The product **17** was obtained as a white powder, yield: 76%, mp 168–170 °C. IR spectrum, *ѵ*, cm^−1^: 3346, 3310, 2928, 2351, 1732, 1616, 1587, 1516, 1259, 1140, 1029, and 794 cm^–1^; ^1^H NMR (400 MHz, CDCl_3_) *δ* 6.99 (t, *J* = 7.9 Hz, 1H, ArH), 6.81 (d, *J* = 7.7 Hz, 1H, ArH), 6.70–6.62 (m, 1H, ArH), 6.62–6.53 (m, 3H, ArH), 4.88 (d, *J* = 6.5 Hz, 1H, CH-N), 4.76 (br. d, *J* = 3.0 Hz, 2H, NH), 3.72 (s, 6H, OCH_3_), 3.69 (s, 3H, OCH_3_), 3.25 (t, *J* = 6.5 Hz, 2H, CH_2_), 2.60 (t, *J* = 6.9 Hz, 2H, CH_2_), 2.54–2.44 (m, 2H, CH_2_), 1.86–1.73 (m, 1H, CH_2_), and 1.72–1.53 (m, 3H, CH_2_). ^13^C NMR (101 MHz, CDCl_3_) *δ* 157.79 (CO), 156.92 (C), 148.91 (C), 147.50 (C), 138.99 (C), 131.82 (C), 126.43 (CH), 126.27 (CH), 120.69 (CH), 120.65 (CH), 112.00 (CH), 111.31 (CH), 107.96 (CH), 55.84 (OCH_3_), 55.75 (OCH_3_), 55.25 (OCH_3_), 48.16 (CH), 41.76 (CH_2_), 36.01 (CH_2_), 30.30 (CH_2_), 22.94 (CH_2_), and 19.28 (CH_2_). Analysis calculated for C_22_H_28_N_2_O_4_: C 68.73; H 7.34; N 7.29. Found, %: C 68.75; H 7.36; N 7.32. *M*: 384.48.

#### 1-(5-Methoxy-1,2,3,4-tetrahydronaphthalen-1-yl)-3-(4-methoxyphenethyl)urea (18)

The product **18** was obtained as a white powder, yield: 75%, mp 150–152 °C. IR spectrum, *ѵ*, cm^−1^: 3296, 2935, 2323, 1614, 1568, 1513, 1467, 1250, 1083, 1039, and 821 cm^–1^; ^1^H NMR (400 MHz, CDCl_3_) *δ* 7.18–7.05 (m, 3H, ArH), 6.93 (d, *J* = 7.8 Hz, 1H, ArH), 6.83 (d, *J* = 8.4 Hz, 2H, ArH), 6.70 (d, *J* = 8.0 Hz, 1H, ArH), 4.97–4.89 (m, 1H, CH-N), 4.48 (br.s, 1H, NH), 4.30 (br.s, 1H, NH), 3.80 (s, 3H, OCH_3_), 3.78 (s, 3H, OCH_3_), 3.40 (t, *J* = 6.7 Hz, 2H, CH_2_), 2.75 (t, *J* = 6.8 Hz, 2H, CH_2_), 2.71–2.52 (m, 2H, CH_2_), 1.99–1.88 (m, 1H, CH_2_), and 1.84–1.70 (m, 3H, CH_2_). ^13^C NMR (101 MHz, CDCl_3_) *δ* 158.19 (C), 157.56 (CO), 156.96 (C), 138.87 (C), 131.11(C), 129.76 (2CH), 126.53 (C), 126.36 (CH), 120.72 (CH), 113.99 (2CH), 108.04 (CH), 55.31 (OCH_3_), 55.26 (OCH_3_), 48.28 (CH), 41.93 (CH_2_), 35.44 (CH_2_), 30.24 (CH_2_), 22.95 (CH_2_), and 19.26 (CH_2_). Analysis calculated for C_21_H_26_N_2_O_3_: C 71.16; H 7.39; N 7.90. Found, %: C 71.20; H 7.42; N 7.87. *M*: 354.45.

### Bacterial strains

The bacteria employed for evaluation included *Acinetobacter baumannii* ATCC 1605, *Enterococcus faecalis* ATCC 49452, *Pseudomonas aeruginosa* ATCC 9027, and *Staphylococcus aureus* MRSA ATCC 43300. These strains were sourced from the Molecular Biology and Genetics Laboratory at Erzurum Technical University. They were immersed in Mueller-Hinton Broth (MHB) and then incubated at a temperature of 37 °C for a duration of 16–24 h.

### Disc diffusion method

Bacteria were adjusted to a McFarland standardisation of 0.5, equivalent to a cell density of 1.5 × 10^8^/ml, and were then introduced into MHB. Subsequently, a 100 μl volume of this bacterial mixture was spread onto Mueller-Hinton agar plates. Sterile empty discs were then treated with the formulated ureas at concentrations of 1000, 500, and 250 µM. Oflaxin (10 g/disc) and netilmicin (30 g/disc) were used as positive controls, while DMSO served as the negative control. The plates were then placed in an incubator at 37 °C for a period ranging from 24 to 72 h, depending on the optimal conditions. Post incubation, the diameter of the inhibition zones was gauged to determine the antibacterial efficacy. This entire process was replicated three times^19^.

### Micro-well dilution assay

The synthesised ureas’ minimum inhibitory concentrations (MICs) against the bacterial samples were ascertained using the microdilution broth technique. Utilising a method of twofold serial dilutions, the ureas were diluted to concentrations spanning from 500 to 0.48 M. The bacterial turbidity was set to match the 0.5 McFarland standard^20^. In the 96-well plates, each well received 100 μl of MHB and 5 μl of the bacteria being tested. For the negative control, 195 μl of MHB and 5 μl of the bacterial sample were added. The plate was then covered with a sterile seal and allowed to incubate for 24 h at a temperature of 37 °C. Post incubation, the growth of the bacteria was assessed at 600 nm using the Fluoroskan Ascent^®^ FL. The MIC represented the smallest amount of the chemical that effectively halted bacterial growth. This procedure was conducted three times^19^.

### Cell line and culture conditions

The cells in culture were frequently sub-cultured, about 2–3 times weekly, until they reached a confluence of 80–90%. The experiments were organised into seven cell groups as follows: group I served as the control, while groups II through VII were pre-treated with varying dosages of the synthesised ureas (ranging from 12.5 µM to 500 µM). The cytotoxic potential of these ureas was assessed *in vitro* using the Cell Viability Detection Kit-8 (CVDK-8, Ecotech Biotechnology, Erzurum, Turkey), a method based on WST-8 quantification for human primary dermal fibroblasts and cervical cancer cells^21^. Healthy fibroblast, HeLa, and SH-SY5Y cells were allocated in 96-well plates, with each well containing 5 × 10^3^ cells in 100 µL of DMEM:F12 (supplemented with 1% penicillin/streptomycin and 10% FBS). After an overnight growth, these cells were exposed to specified concentrations of the synthesised ureas for a duration of 48 h at 37 °C in a 5% CO_2_ atmosphere. Cisplatin acted as the positive control with concentration gradients of 0–250 µM for HeLa cells and 0–20 µM for the SH-SY5Y cells. Post incubation, in sterile, dimly lit conditions, 10% WST-8 solution was added to every well and further incubated at 37 °C within 5% CO_2_ for another 3–4 h. The optical density of the 96-well plate was gauged at a 450 nm wavelength using a spectrophotometer (BioTek, EPOCH, Santa Clara, CA). The control group’s main purpose was to measure the absorbance of just the cells and the growth medium.

### Selectivity index (SI)

This research denotes the selectivity level of the created compounds as follows: SI is calculated by dividing the IC_50_ value of the unadulterated compound in the standard cell line by the IC_50_ value of the same compound in the cancerous cell line. Here, IC_50_ represents the concentration required to eradicate 50% of the cell community^22^.

### Statistical analysis

In analytical statistics, all of the experiments were carried out in triplets. All analyses were carried out using the GraphPad Prism program (GraphPad Software, La Jolla, CA), and the findings were given as mean ± standard deviation.

## Results

**Scheme 1.**
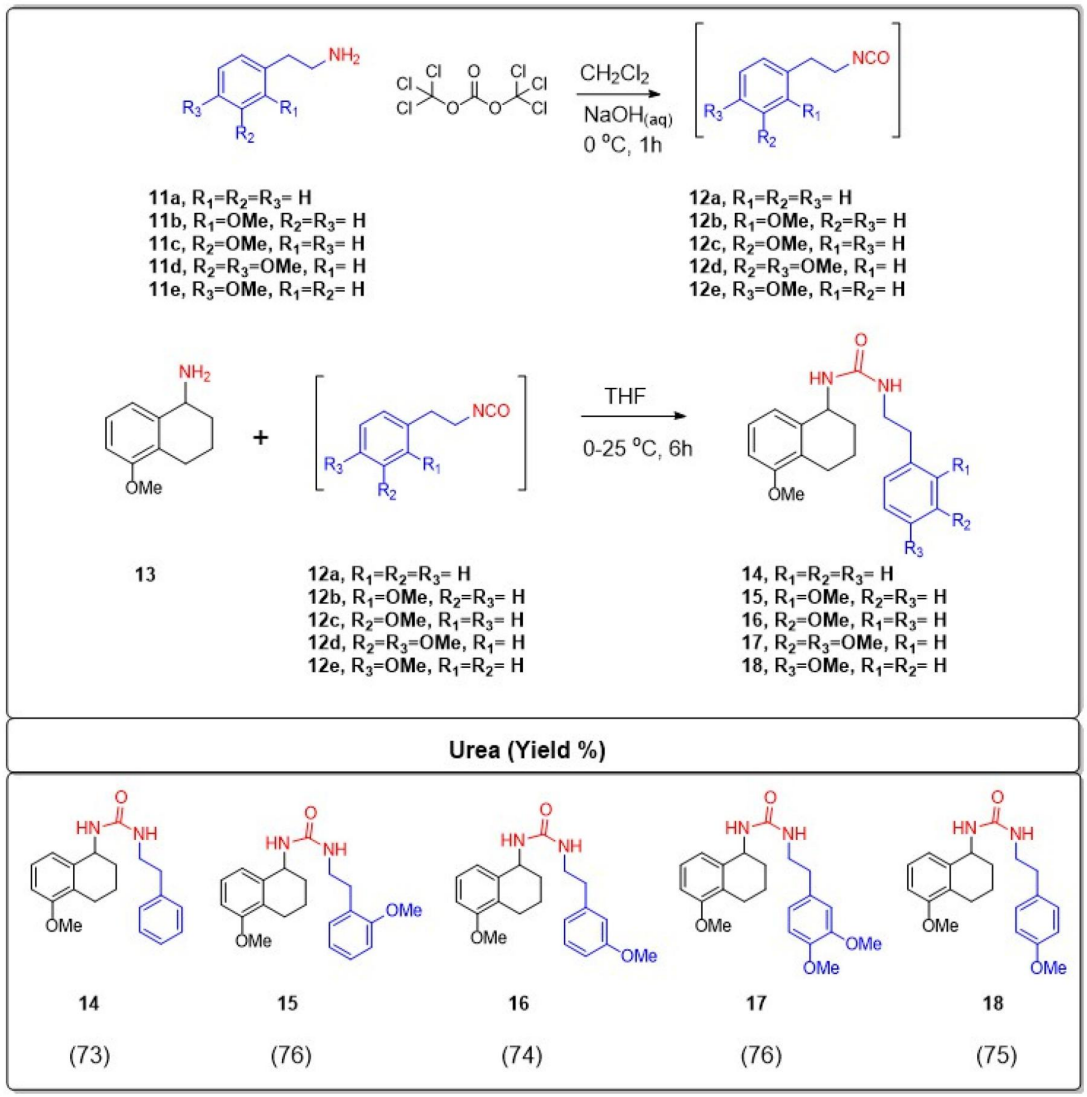
Synthesis of substituted tetrahydronaphthalen-1-yl-phenethyl ureas **14–18**.

In the initial phase of the synthesis, phenethylamines, which are a class of organic compounds that contain a phenyl ring attached to an amino group, were designated as **11a–e** based on variations in their molecular structures or substituents. These were subjected to a reaction with triphosgene. Triphosgene is an organic compound often used as a more convenient substitute for phosgene, as it releases phosgene upon heating. In the context of this reaction, triphosgene serves as a reagent to convert the phenethylamine derivatives **11a–e** into isocyanate intermediates. Isocyanates are functional groups containing the –N═C═O moiety. The resulting isocyanate intermediates were labelled as compounds **12a–e**. Without undergoing any further purification, these isocyanate intermediates (**12a–e**) were immediately employed in the subsequent stage of the synthesis. 5-Methoxy-1-aminotetralin, which is labelled as compound **13**, possesses an amine group (–NH2). This compound was reacted with the aforementioned isocyanate intermediates **12a–e**. The choice of solvent for this reaction was tetrahydrofuran (THF), which is a polar aprotic solvent commonly used in organic synthesis. The reactions were carried out under specific temperature conditions, ranging from 0 °C to 25 °C. Under these conditions, the amine group from the 5-methoxy-1-aminotetralin 13 undergoes a nucleophilic attack on the electrophilic carbon of the isocyanate group, leading to the formation of N,N′-dialkyl ureas. Ureas are compounds with the functional group –NH–CO–NH–, and in this case, they are dialkylated, implying the presence of two alkyl groups attached to the nitrogen atoms. The resultant novel N,N′-dialkyl ureas were denoted as compounds **14–18**. The overall efficiency of this synthesis is commendable, with high yields reported between 73% and 76% (Scheme 1).

All the synthesised compounds had their chemical structures defined using elemental analysis, IR, ^1^H NMR, and ^13^C NMR. In the ^1^H NMR spectrum for N,N′-dialkyl ureas labelled **14–18**, the protons related to the HN(CO)NH showed either as a broad singlet or broad doublet within the range of 4.76–4.30 ppm. For the same ureas, the OCH_3_ proton signals were observed as singlets between 3.81 and 3.66 ppm. Meanwhile, in the ^13^C NMR spectrum for these ureas, the –CO– signals were detected between 157.75 and 157.56 ppm, while the OCH_3_ signals ranged from 55.84 to 55.10 ppm.

The antibacterial properties of the formulated ureas, labelled **14–18**, were evaluated against various microorganisms. This included two types of Gram-negative bacteria (namely *Acinetobacter baumannii* and *Pseudomonas aeruginosa*) and two strains of Gram-positive bacteria (specifically *Enterococcus faecalis* and *Staphylococcus aureus* MRSA) using the disc diffusion method. The outcomes of the antibacterial activity, along with the MIC values for these ureas, can be found in Table 1.

**Table 1.** Antibacterial activity of the synthesised ureas **14–18**.

Bacteria	Number of ureas	Disc diffusion test^a^ Concentrations (µM)			MIC^b^ (µM)	Negative control	Standard antibiotic discs^c^	
		1000	500	250		DMSO	OFX	NET30
*Acinetobacter baumannii*	14	–	–	–	–	–	16 MIC: 0.5 µg/ml	13 MIC: 4 µg/ml
	15	–	–	–	–			
	16	13	10	5	7.8125			
	17	15	8	6	7.8125			
	18	16	10	6	7.8125			
*Enterococcus faecalis*	14	–	–	–	–	–	11 MIC: 2.5 µg/ml	9 MIC: 32 µg/ml
	15	–	–	–	–			
	16	7	6	–	3.90			
	17	10	7	–	0.97			
	18	13	7	5	0.97			
*Pseudomonas aeruginosa*	14	–	–	–	–	–	23 MIC: 11.4 µg/ml	16 MIC: 4 µg/ml
	15	5	5	5	15.625			
	16	10	8	7	7.8125			
	17	10	10	7	7.8125			
	18	15	10	9	3.90			
*Staphylococcus aureus MRSA*	14	–	–	–	–	–	24 MIC: 1.2 µg/ml	21 MIC: 0.8 µg/ml
	15	6	5	–	15.625			
	16	13	6	5	7.8125			
	17	14	13	8	7.8125			
	18	15	12	5	3.90			

^a^Inhibitory zone diameter (mm) varies based on different concentrations.

^b^MIC: minimum inhibitory concentrations.

^c^OFX refers to ofloxacin at a concentration of 10 μg/disc, while NET-30 indicates netilmycin at 30 μg/disc. Both were utilised as benchmark antibiotic discs from Oxoid.

The WST-8 test was conducted on SH-SY5Y, HeLa, and PCS-201-012 cells after being treated with increasing concentrations of the produced compounds for 48 h to determine their cytotoxic effects. In this study, we used two different chemotherapeutic drugs as controls. One of them is cisplatin (cis-diammineplatinum(II) dichloride), which is a widely used antineoplastic drug for clinical cures and was used as a positive control (IC_50_ value for SH-SY5Y cell line: 10 µM^23^, for HeLa cell line: >50 µM^24^, and for fibroblast cells: >100 µM^25^). Another control is melphalan, which is also a chemotherapy drug. It is used as a treatment for several different cancer types. The IC_50_ value for the HeLa cell is 61 µM^26^, while it is 2.2 µM^27^ for the SH-SY5Y cell. The synthesised ureas **14–18** were treated to SH-SY5Y, HeLa, and PCS-201-012 cells for 48 h at concentrations of 12.5, 25, 50, 100, 300, and 500 M. Table 2 shows the IC_50_ values for several compounds.

**Table 2.** Cytotoxic activity (IC_50_, μM) of the synthesised ureas (*n* = 4).

Test molecules	SH-SY5Y	HeLa cells	PCS-201-012 cells
14	9.7	2.6	7.9
15	8.9	142	225.7
16	19.1	32.4	>500
17	9.7	251	379.8
18	10.2	364.4	373.5
Cisplatin	10	75 > IC_50_ > 50	>100
Melphalan	2.2	61	–

**Figure 3.**
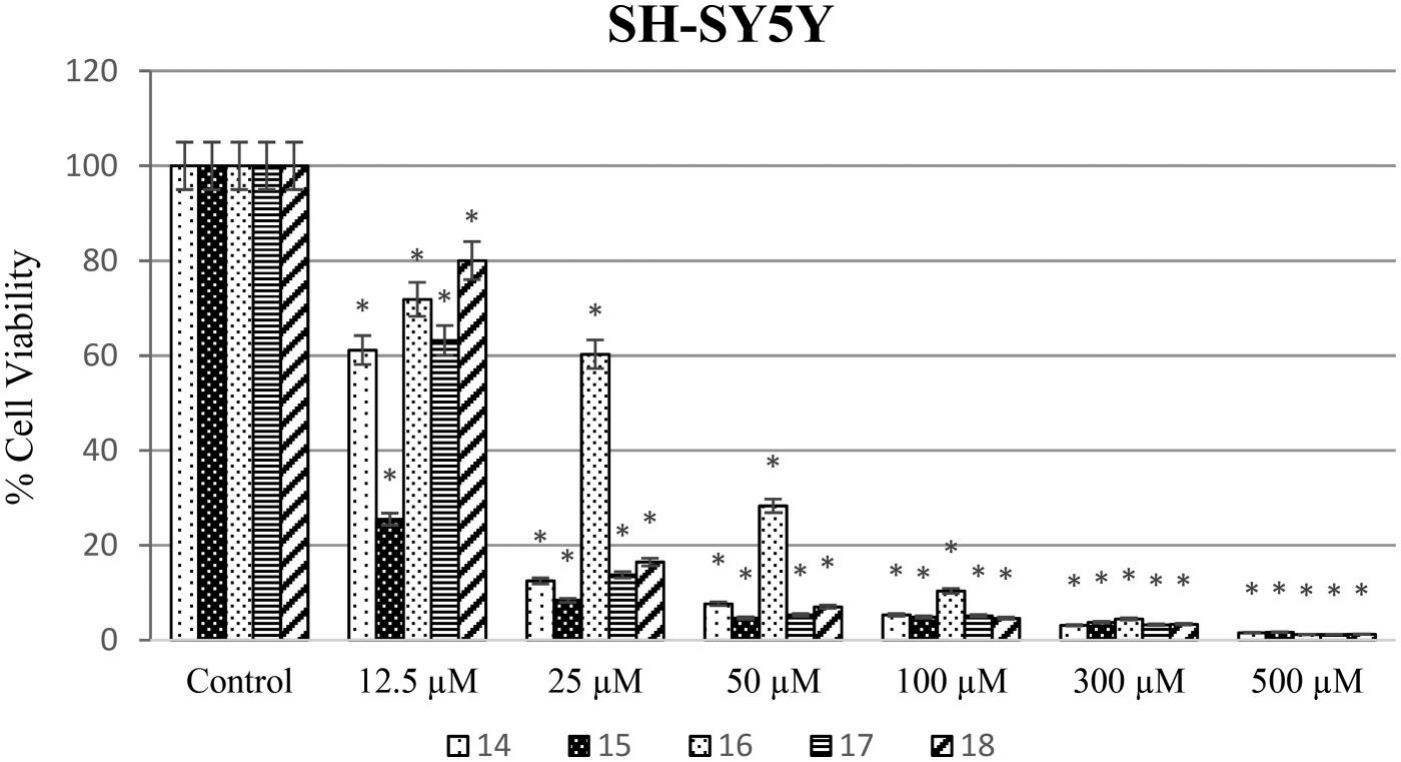
SH-SY5Y cells were exposed to produced ureas (12.5–500 M) for 48 h, and cytotoxicity was assessed using the WST-8 assay. GraphPad Prism 7.00 (GraphPad Software, La Jolla, CA) was used for statistical analysis, and ANOVA: Dunnett’s multiple comparison test calculated the results. *p < 0.01 when compared to the control group.

**Figure 4.**
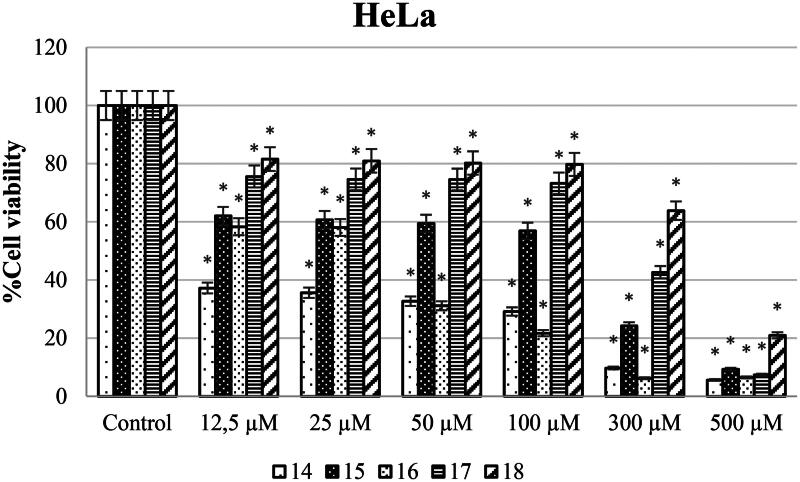
HeLa cells were exposed to produced ureas (12.5–500 M) for 48 h, and cytotoxicity was assessed using the WST-8 assay. GraphPad Prism 7.00 (GraphPad Software, La Jolla, CA) was used for statistical analysis, and ANOVA: Dunnett’s multiple comparison test calculated the results. *p < 0.01 when compared to the control group.

Different concentrations of ureas (from 12.5 to 500 µM) were applied to SH-SY5Y, HeLa, and PCS-201-012 cells for 48 h. As shown in Figure 3, SH-SY5Y cells exposed to compound 14 at concentrations of 12.5, 25, 50, 100, 300, and 500 µM showed the lowest cell viability rates of 25.48, 8.40, 4.68, 4.87, 3.77, and 1.67%, respectively. In addition, cell viability varies (58.3, 58, 31.1, 21.7, 6.4, and 6.4%) when HeLa cell lines were treated with newly synthesised ureas at concentrations of 12.5, 25, 50, 100, 300, and 500 µM (Figure 4).

Anticarcinogenic analyses of the synthesised molecules on the SH-SY5Y cell line showed that compound **15** has the highest anticancer potential compared to other molecules. On the other hand, compound **16** was investigated to have the lowest anticarcinogenic property against the neuroblastoma cell line (Figure 4). Besides, anticancer potential of synthesised molecules was found higher against the SH-SY5Y cell line compared to the HeLa cell line for all of the compounds. In parallel to the literature, it was shown in different studies that same anticarcinogen could exhibit diverse cytotoxic efficiency against different cancer cell types^28^^,^^29^. Drug formulations should be chosen and modified according to tumour type and the molecular structures should be decided according to the target cell line.

Also, when examining the IC_50_ values of the synthesised compounds against the HeLa cell line, urea derivatives **14** and **16** had the best anticancer activity. However, urea derivative **18** had lower anticancer activity against the HeLa cell line. In our previous study, we reported that while urea derivative containing 7-methoxy-1-aminotetralin has the best activity with has 58.9 µM IC_50_ value against HeLa cell line^17^. On the other hand, we have shown that our compounds **14** (2.6 µM) and **16** (32.4 µM) have IC_50_ values against HeLa cell line in this study. When looking at this aspect, we can say that compounds containing 5-methoxy-1-aminotetralin have the best activity against cancer cell lines. In addition, Özgeris reported that urea derivatives containing substituted phenethylamine’s structure showed strong anticancer activity against HeLa cell lines^30^, which supports our study.

**Figure 5.**
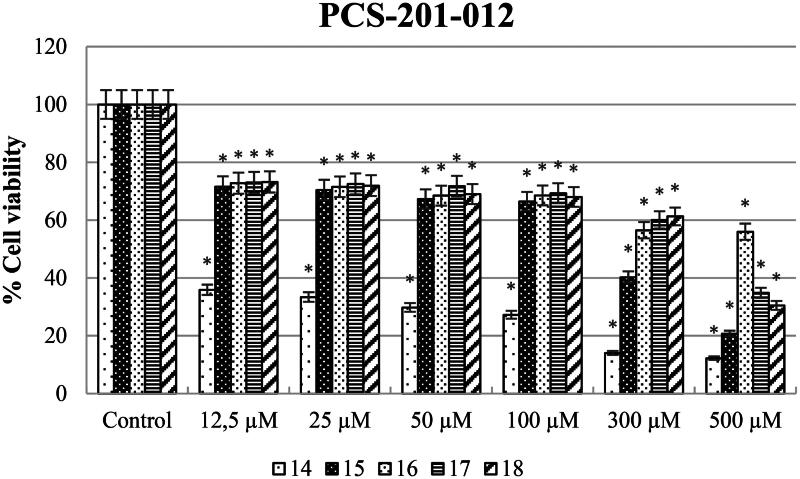
Percentage of cytotoxicity evaluated using the WST-8 test on PCS-201-012 cells after 48 h of exposure to produced ureas 14–18 (12.5–500 M). GraphPad Prism 7.00 (GraphPad Software, La Jolla, CA) was used for statistical analysis, and ANOVA: Dunnett’s multiple comparison test calculated the results. *p < 0.01 when compared to the control group.

On the other hand, the cytotoxic effect of compounds **14–18** against PCS201-012 cell lines was also assessed (Figure 5). According to the results, compound **15** (IC_50_ on PCS201-012: 225.7 µM) was up to twofold more potent than cisplatin against the HeLa cell line while less toxic than cisplatin (IC_50_ on PCS201-012: 100 µM) against the PCS201-012 cell line. Also, compound **16**'s anticancer activity exhibited a low toxic effect against healthy cell lines compared to cisplatin treatment (IC_50_ on PCS201-012: 100 µM).

In addition, we can say that all synthesised compounds except compound **14** have fewer toxic effects on healthy cell lines when compared to cisplatin (Table 2).

Selectivity index value was calculated by dividing the IC_50_ value of non-cancerous human dermal fibroblast cells with the IC_50_ value of the cancer cell lines (Table 3).

**Table 3.** SI values of the synthesised molecules (*n* = 4).

Test molecules	SI value (SH-SY5Y cells)	SI value (HeLa cells)
14	0.81	3.04
15	25.26	1.58
16	26.10	15.4
17	39.08	1.51
18	36.72	1.02

According to the results, all compounds except 14 had at least 25-fold less cytotoxic effect for the SH-SY5Y cell line. The most effective compound is 18 with 36.72-fold. For the HeLa cell line, compound **14** up to threefold (SI: 3.04), compound **16** exhibited up to 15-fold less toxic effect (SI: 15.4). Suffness reported that derivatives exhibiting an SI value >2 were accepted as selective^31^. This report supports that our results have the best toxicity profiles in the aspect of the SI. Therefore, all urea derivatives except for compound **14** can be considered as anticancer agents for SH-SY5Y and urea derivatives **14** and **16** can be considered against HeLa cancer.

## Discussion

A number of experimental methodologies were developed to produce urea derivatives such as using phosgene or triphosgene functionalisation^32^, addition of amine to isocyanate^32^, 1,1-carbonyldiimidazole (CDI) coupling^33^, carbonylimidazolium salt^34^, dialkyl-based exchange processes^35^, oxidative carbonylation of amines^36^, and derivatisation of chloroformate^37^. Isocyanates play a pivotal role as precursor intermediates in the creation of biologically significant compounds. Alkyl isocyanates enable the straightforward production of unsymmetrical ureas. A predominant technique for isocyanate synthesis involves reacting amines with phosgene. Nonetheless, handling and storing phosgene poses challenges due to its toxic gaseous nature^38^. Therefore, the use of phosgene in the laboratory is disadvantageous. Triphosgene (bis(trichloromethyl) carbonate) is easy to store and transport since it is a crystalline, stable solid^39^. Therefore the most frequent alternative to phosgene for the production of isocyanates is triphosgene^40^. Hence, phenethylamines **11a–e** were reacted with triphosgene to give isocyanate intermediates **12a–e**. Compounds **12a–e** were used in the next step without further purification. The reactions of 5-methoxy-1-aminotetralin **13** with isocyanates **12a–e** in THF at 0–25 °C yielded novel N,N′-dialkyl ureas **14–18** in high yields (73–76%).

The antibacterial properties of the formulated ureas, particularly those labelled as **14–18**, have shown significant potential in combating a range of bacterial pathogens. The Gram-negative bacteria, especially *Acinetobacter baumannii* and *Pseudomonas aeruginosa*, have historically been associated with various resistant infections, particularly in healthcare settings^41^. Similarly, the Gram-positive strains, *Enterococcus faecalis* and *Staphylococcus aureus* MRSA, are notorious for their virulence and multidrug resistance profiles^42^. The evaluation of these ureas against such formidable bacterial adversaries using the disc diffusion method provides a comprehensive understanding of their potential applications in the therapeutic landscape. The inclusion of MIC values in Table 1 further elucidates the potency of these compounds in inhibiting bacterial growth. With the global increase in antibiotic-resistant bacterial strains, the discovery and assessment of such compounds become invaluable^43^.

Urea compound **14** does not have any modifications on its phenethylamine ring, while compound **18** is characterised by a methoxy group positioned at the para location on its phenethylamine ring. When evaluating their MIC values alongside their molecular structures, compound **18** stood out, showing values between 0.97 and 15.625 µM against all bacterial strains tested. On the other hand, urea compound **14** did not display any antibacterial properties against the studied bacteria. Interestingly, a prior study suggested that thiourea variants with methoxy groups on the phenethylamine ring often have diminished antibacterial capabilities^44^. Contrary to that study, we found that urea derivatives with a methoxy group at the para position on the phenethylamine ring displayed significantly potent antibacterial properties. Furthermore, a recent study highlighted that thiourea compounds with a fluorine group in the same para position on the phenethylamine ring showed only moderate antibacterial effects^30^. Another study found that benzoyl thiourea compounds with a para methoxy aniline group have lower antibacterial activity (MIC: generally >1000 M) than standard drugs^45^. As it is seen that, urea compounds having a methoxy group on the phenethylamine ring demonstrate strong antibacterial activity, while thiourea compounds containing a methoxy group on a similar structure demonstrate lower antibacterial activity. This may be explained by the presence of the CO group. The remaining urea compounds **15–18** showed strong to moderate antibacterial activity against all tested bacterial strains except compound **15**.

The evaluation of ureas across varying concentrations provides essential insights into their potential as anticarcinogenic agents, particularly against specific cell lines. The SH-SY5Y cell line, which represents a model for neuroblastoma cells, demonstrated a significant reduction in cell viability upon exposure to compound **14**, suggesting a potent cytotoxic effect^46^. The viability rates observed in SH-SY5Y cells were substantially lower compared to those in the HeLa cell line when exposed to the same urea concentrations. This could hint at a targeted or selective cytotoxic action of these ureas towards neuroblastoma cells over cervical cancer cells (i.e. HeLa)^47^. Interestingly, compound **15** stood out as the most potent anticarcinogenic molecule against SH-SY5Y, while compound **16** exhibited the least efficacy. Such variations in the anticancer potential of individual urea compounds might be attributed to differences in their chemical structures and how they interact with cellular mechanisms^48^. The notable differential cytotoxic effects between the SH-SY5Y and HeLa cell lines underscore the importance of screening potential anticancer agents across diverse cell types to ascertain specificity and potency.

Cisplatin and melphalan are FDA-approved and are among the most crucial chemotherapy drugs used today for cancer treatment. For a drug to be approved by the FDA, it must meet specific safety criteria. Considering this information, we see that the effects of the newly synthesised urea derivatives on SH-SY5Y cells are generally in parallel with the IC_50_ value of cisplatin when compared with cisplatin. In addition, the effects of newly synthesised compounds on healthy cells should be examined to be predicted as a new candidate drug. For this, when the SI values are examined, it is seen that the others, except for compound number **14**, have a very low cytotoxic effect against healthy cells. The IC_50_ values of cisplatin and melphalan used as controls were roughly the same on the HeLa cell line. Considering this value, it can be said that compounds **14** and **16**, newly synthesised compounds, are more effective for HeLa.

Recent studies have converged on the therapeutic potential of urea and phenethylamine derivatives, positioning them as potent dual inhibitors of enzymes pivotal in the fight against cancer and bacterial infections. Urea derivatives have shown promising results as topoisomerase inhibitors, with compounds like benzimidazole urea derivatives emerging as dual inhibitors capable of targeting DNA topoisomerase II and IV, essential in cancer cell replication. Notably, the development of hexacyclic camptothecin analogues containing urea groups has highlighted their strong antitumor activities, challenging the efficacy of established chemotherapeutic agents^49^. Concurrently, tetrahydrobenzothieno[2,3-d]pyrimidine urea derivatives have demonstrated superior anticancer efficacy, offering new avenues for oncological drug development^50^^,^^51^. In parallel, phenethylamine derivatives, traditionally linked to neurotransmitter modulation, are being explored for their inhibitory action on DNA gyrase, an enzyme integral to bacterial DNA replication. Although research is in the early stages, the application of structure–activity relationship analysis and molecular docking suggests that phenethylamine derivatives could serve as a novel class of antimicrobial agents^52^^,^^53^. This exploration is especially critical given the rise of antimicrobial resistance, which presents similar challenges to those of drug-resistant cancers^54^^,^^55^. Integrating insights from recent research, both urea and phenethylamine derivatives are emerging as multifaceted molecules with the potential to disrupt DNA processes critical to cancerous and bacterial cell proliferation, marking a significant stride in the development of new anticancer and antibacterial therapies. The convergence of anticancer and antibacterial properties within the same therapeutic agent is an area of significant interest in drug development. The synthesised molecules, which have been designed to possibly target critical enzymes involved in DNA replication and repair, showcase this duality of therapeutic action. These compounds likely owe their efficacy to a mechanism involving the inhibition of topoisomerase enzymes. By inhibiting these enzymes, our molecules induce DNA damage that ultimately leads to cell death in both bacterial and cancer cells. In bacterial cells, the synthesised molecules appear to target DNA gyrase and topoisomerase IV – key enzymes required for bacterial DNA replication. The inhibition of these enzymes prevents the supercoiling necessary for proper replication, leading to bacterial cell death. This mode of action is reminiscent of that utilised by fluoroquinolones, a well-established class of antibiotics^56^. Conversely, in cancer cells, the same molecules exhibit the ability to interfere with topoisomerase II, a pivotal enzyme in DNA replication and repair in eukaryotic cells. The inhibition of topoisomerase II disrupts the replication process and can trigger apoptosis in rapidly dividing cancer cells, similar to the action of chemotherapeutic agents such as etoposide^57^. Notably, this topoisomerase II inhibition characteristic has been evidenced in our molecular compounds, which have demonstrated significant anti-proliferative activity against a range of cancer cell lines. This dual inhibition underscores the potential versatility and wide-ranging applicability of our synthesised compounds. The anticancer and antibacterial activities of our synthesised molecules, stemming from their topoisomerase inhibition mechanism, present a unique therapeutic opportunity. They could potentially streamline treatment protocols by addressing both bacterial infections and cancerous growths concurrently, particularly in clinical scenarios where bacterial infections complicate cancer progression. Furthermore, these dual-action inhibitors may provide an alternative pathway to combat antibiotic resistance in bacteria and resistance to chemotherapy in cancer cells, a significant concern in both fields^58^^,^^59^. In essence, the dual pharmacological profiles of our molecules not only position them as promising candidates for further development but also underscore the importance of topoisomerase as a target for designing versatile and potent new drugs for complex diseases^60^^,^^61^.

## Conclusions

As a result, the resistance developed by cancer patients against chemotherapeutic drugs and the resistance developed by people against antibiotics have led scientists to find new drugs. It has been shown by previous studies that urea derivatives are the best candidates for this. Although the cytotoxic and antibacterial activities of many urea compounds have been investigated, the biological activities of our newly synthesised urea compounds against cancer cells or bacteria have not been investigated before. A series of substituted phenethylamine-based N,N′-dialkyl urea derivatives containing 1-aminotetralin and carrying methoxy substituents at various sites of phenethylamine were synthesised within the scope of this project. Synthesis of N,N′-dialkyl urea derivatives **14–18** was performed for the first time in good yields (73–76%). According to the antibacterial results, all tested compounds showed potent activity against all tested bacterial strains, except for compound **14** (MIC values: 0.97–15.82 µM). In anticancer activity against the HeLa cell line, urea derivatives **14** (IC_50_: 2.6 µM) and **16** (IC_50_: 32.4 µM) exhibited the best activity. In addition, the anticancer activities of the synthesised compounds showed a highly effective anticarcinogenic potential on the SH-SY5Y cell line. In particular, compound **15** exhibited the highest anticancer activity with an IC_50_ of 8.93 µM.

Moreover, the tested urea derivatives showed reasonable safety against the normal human dermal fibroblast cell line PCS201-012. On the other hand, it was observed that compounds **16**, **17**, and **18** were the most potent antimicrobial and anticarcinogenic candidate molecules. Also, among these compounds, molecule **16** has been shown to have the highest anticancer activity against the HeLa cell line, and compounds **17** and **18** exhibit the highest anticarcinogenic activity against the SH-SY5Y cell line. All newly synthesised compounds for the SH-SY5Y cell line were found to have the same cytotoxic effect as the average cisplatin compared to cisplatin used as a positive control. When the HeLa cell line is examined, it can be said that compounds **14** and **16** are more effective than cisplatin. Finally, looking at the cytotoxic effect on the healthy cell line, all seem to be less toxic than cisplatin, except for compound **14**. Considering the results, it is predicted that the compounds can be used as anticancer and antibacterial drug candidate, but it needs to be developed with additional pharmacokinetic studies.

## Data Availability

The data presented in this study are available on request from the corresponding author. The data are not publicly available due to privacy.

## References

[1] Zhang Y, Anderson M, Weisman JL, Lu M, Choy CJ, Boyd VA, Price J, Sigal M, Clark J, Connelly M, et al. Evaluation of diarylureas for activity against *Plasmodium falciparum*. ACS Med Chem Lett. 2010;1(9):460–465. 21243104 10.1021/ml100083c PMC3019604

[2] Paget CJ, Kisner K, Stone RL, DeLong DC. Heterocyclic substituted ureas. II. Immunosuppressive and antiviral activity of benzothiazolyl- and benzoxazolylureas. J Med Chem. 1969;12(6):1016–1018. 5351441 10.1021/jm00306a011

[3] Schroeder MC, Hamby JM, Connolly CJ, Grohar PJ, Winters RT, Barvian MR, Moore CW, Boushelle SL, Crean SM, Kraker AJ, et al. Soluble 2-substituted aminopyrido[2,3-d]pyrimidin-7-yl ureas. Structure–activity relationships against selected tyrosine kinases and exploration of in vitro and in vivo anticancer activity. J Med Chem. 2001;44(12):1915–1926. 11384237 10.1021/jm0004291

[4] Wróbel TM, Kiełbus M, Kaczor AA, Kryštof V, Karczmarzyk Z, Wysocki W, Fruziński A, Król SK, Grabarska A, Stepulak A, et al. Discovery of nitroaryl urea derivatives with antiproliferative properties. J Enzyme Inhib Med Chem. 2016;31(4):608–618. 26114307 10.3109/14756366.2015.1057716

[5] North EJ, Scherman MS, Bruhn DF, Scarborough JS, Maddox MM, Jones V, Grzegorzewicz A, Yang L, Hess T, Morisseau C, et al. Design, synthesis and anti-tuberculosis activity of 1-adamantyl-3-heteroaryl ureas with improved in vitro pharmacokinetic properties. Bioorg Med Chem. 2013;21(9):2587–2599. 23498915 10.1016/j.bmc.2013.02.028 PMC3780361

[6] Pandurangan K, Kitchen JA, Blasco S, Paradisi F, Gunnlaugsson T. Supramolecular pyridyl urea gels as soft matter with antibacterial properties against MRSA and/or *E. coli*. Chem Commun. 2014;50(74):10819–10822. 10.1039/c4cc04028g 25089301

[7] Hofmann C, Penner U, Dorow R, Pertz HH, Jähnichen S, Horowski R, Latté KP, Palla D, Schurad B. Lisuride, a dopamine receptor agonist with 5-HT2B receptor antagonist properties: absence of cardiac valvulopathy adverse drug reaction reports supports the concept of a crucial role for 5-HT2B receptor agonism in cardiac valvular fibrosis. Clin Neuropharmacol. 2006;29(2):80–86. 16614540 10.1097/00002826-200603000-00005

[8] Reichmann H. Long-term treatment with dopamine agonists in idiopathic in Parkinson’s disease. J Neurol. 2000;247(4):IV17–IV19. 10.1007/pl00007768 11199810

[9] Zeldin RK, Petruschke RA. Pharmacological and therapeutic properties of ritonavir-boosted protease inhibitor therapy in HIV-infected patients. J Antimicrob Chemother. 2004;53(1):4–9. 14657084 10.1093/jac/dkh029

[10] Igwe KK, Ikpeazu OV, Otuokere IE, Department of Veterinary Biochemistry and Animal Production, Michael Okpara University of Agriculture, Umudike, Nigeria. Global energy and molecular interactions between pazopanib, axitinib and sorafenib anticancer drugs with vascular endothelial growth factor. J Biosci Biotechnol Discov. 2017;2(4):91–96.

[11] Matsui J, Funahashi Y, Uenaka T, Watanabe T, Tsuruoka A, Asada M. Multi-kinase inhibitor E7080 suppresses lymph node and lung metastases of human mammary breast tumor MDA-MB-231 via inhibition of vascular endothelial growth factor-receptor (VEGF-R) 2 and VEGF-R3 kinase. Clin Cancer Res. 2008;14(17):5459–5465. 18765537 10.1158/1078-0432.CCR-07-5270

[12] Sabelli HC, Mosnaim AD, Vazquez AJ, Giardina WJ, Borison RL, Pedemonte WA. Biochemical plasticity of synaptic transmission: a critical review of Dale’s principle. Biol Psychiatry. 1976;11(4):481–524. 9160

[13] Finberg JPM, Gillman K. Selective inhibitors of monoamine oxidase type B and the “cheese effect”. Int Rev Neurobiol. 2011;100:169–190. 21971008 10.1016/B978-0-12-386467-3.00009-1

[14] Carlsson A. Biochemical and pharmacological aspects of Parkinsonism. Acta Neurol Scand Suppl. 1972;51:11–42. 4351191

[15] Bredikhina ZA, Kurenkov AV, Krivolapov DB, Bredikhin AA. Synthesis of all of the stereoisomers of β3-adrenoceptor antagonist SR 59230 based on the spontaneous resolution of 3-(2-ethylphenoxy) propane-1,2-diol. Tetrahedron Asymmetry. 2016;27(11–12):467–474.

[16] Welch WM, Kraska AR, Sarges R, Koe BK. Nontricyclic antidepressant agents derived from cis- and trans-1-amino-4-aryltetralins. J Med Chem. 1984;27(11):1508–1515. 6492080 10.1021/jm00377a021

[17] Akbaba Y, Kacı FN, Göksu S. Substituted tetrahydronaphthalen‐1‐yl‐phenethyl ureas: synthesis, characterization, and biological evaluations. ChemistrySelect. 2022;7(15):e202200450.

[18] Özgeriş B, Akbaba Y, Özdemir Ö, Türkez H, Göksu S. Synthesis and anticancer activity of novel ureas and sulfamides incorporating 1-aminotetralins. Arch Med Res. 2017;48(6):513–519. 29248174 10.1016/j.arcmed.2017.12.002

[19] Gormez A, Bozari S, Yanmis D, Gulluce M, Sahin F, Agar G. Chemical composition and antibacterial activity of essential oils of two species of Lamiaceae against phytopathogenic bacteria. Pol J Microbiol. 2015;64(2):121–127. 26373171

[20] Wiegand I, Hilpert K, Hancock REW. Agar and broth dilution methods to determine the minimal inhibitory concentration (MIC) of antimicrobial substances. Nat Protoc. 2008;3(2):163–175. 18274517 10.1038/nprot.2007.521

[21] Ishiyama M, Miyazono Y, Sasamoto K, Ohkura Y, Ueno K. A highly water-soluble disulfonated tetrazolium salt as a chromogenic indicator for NADH as well as cell viability. Talanta. 1997;44(7):1299–1305. 18966866 10.1016/s0039-9140(97)00017-9

[22] Koch A, Tamez P, Pezzuto J, Soejarto D. Evaluation of plants used for antimalarial treatment by the Maasai of Kenya. J Ethnopharmacol. 2005;101(1–3):95–99. 15878245 10.1016/j.jep.2005.03.011

[23] Yang C, Tan J, Zhu J, Wang S, Wei G. YAP promotes tumorigenesis and cisplatin resistance in neuroblastoma. Oncotarget. 2017;8(23):37154–37163. 28415761 10.18632/oncotarget.16209 PMC5514898

[24] Sasaki-Kudoh E, Kudo I, Kakizaki Y, Hosaka M, Ikeda S-I, Uemura S, Grave E, Togashi S, Sugawara T, Shimizu H, et al. Cisplatin inhibits AhR activation. Am J Mol Biol. 2018;8(1):69–82.

[25] Czarnomysy R, Bielawski K, Muszynska A, Bielawska A, Gornowicz A. Biological evaluation of dimethylpyridine–platinum complexes with potent antiproliferative activity. J Enzyme Inhib Med Chem. 2016;31(Suppl. 3):150–165. 27488500 10.1080/14756366.2016.1212191

[26] Mphahlele MJ, Gildenhuys S, Parbhoo N. Synthesis, cytotoxicity and molecular docking studies of the 9-substituted 5-styryltetrazolo [1, 5-c]quinazoline derivatives. Molecules. 2017;22(11):1719. 29072619 10.3390/molecules22111719 PMC6150304

[27] Xu H, Cheung IY, Wei XX, Tran H, Gao X, Cheung NV. Checkpoint kinase inhibitor synergizes with DNA-damaging agents in G 1 checkpoint-defective neuroblastoma. Int J Cancer. 2011;129(8):1953–1962. 21154747 10.1002/ijc.25842

[28] Shao JW, Dai YC, Xue JP, Wang JC, Lin FP, Guo YH. In vitro and in vivo anticancer activity evaluation of ursolic acid derivatives. Eur J Med Chem. 2011;46(7):2652–2661. 21514015 10.1016/j.ejmech.2011.03.050

[29] Zargan J, Sajad M, Umar S, Naime M, Ali S, Khan HA. Scorpion (*Androctonus crassicauda*) venom limits growth of transformed cells (SH-SY5Y and MCF-7) by cytotoxicity and cell cycle arrest. Exp Mol Pathol. 2011;91(1):447–454. 21536027 10.1016/j.yexmp.2011.04.008

[30] Özgeriş B. Synthesis of substituted phenethylamine-based thioureas and their antimicrobial and antioxidant properties. Russ J Org Chem. 2021;57(3):422–429.

[31] Suffness M, Pezzuto JM. Assays related to cancer drug discovery. In: Hostettmann, editor. Methods in plant biochemistry: assays for bioactivity. Vol. 6. London (UK): Academic Press; 1990. p. 71–133.

[32] McMorris TC, Chimmani R, Alisala K, Staake MD, Banda G, Kelner MJ. Structure–activity studies of urea, carbamate, and sulfonamide derivatives of acylfulvene. J Med Chem. 2010;53(3):1109–1116. 20067264 10.1021/jm901384s

[33] Duspara PA, Islam MS, Lough AJ, Batey RA. Synthesis and reactivity of N-alkyl carbamoylimidazoles: development of N-methyl carbamoylimidazole as a methyl isocyanate equivalent. J Org Chem. 2012;77(22):10362–10368. 23083426 10.1021/jo302084a

[34] Grzyb JA, Shen M, Yoshina-Ishii C, Chi W, Brown RS, Batey RA. Carbamoylimidazolium and thiocarbamoylimidazolium salts: novel reagents for the synthesis of ureas, thioureas, carbamates, thiocarbamates and amides. Tetrahedron. 2005;61(30):7153–7175.

[35] Tundo P, Selva M. The chemistry of dimethyl carbonate. Acc Chem Res. 2002;35(9):706–716. 12234200 10.1021/ar010076f

[36] Diaz DJ, Darko AK, McElwee‐White L. Transition metal‐catalyzed oxidative carbonylation of amines to ureas. Eur J Org Chem. 2007;2007(27):4453–4465.

[37] Ready JM, Nijhawan D, Gonzales SS, Theodoropoulos P. Preparation of benzothiophenes and other compounds and methods and compositions for selective and targeted cancer therapy. PCT Int Appl. 2015; WO2015035051A1. 307 pp.

[38] Eckert H, Forster B. Triphosgene, a crystalline phosgene substitute. Angew Chem Int Ed Engl. 1987;26(9):894–895.

[39] Shi H, Hu W, Sun Y. Preparation of chloroformates using bis(trichloromethyl)carbonate. J Chem Res. 2004;2004(10):708–709.

[40] Rose TE, Morisseau C, Liu J-Y, Inceoglu B, Jones PD, Sanborn JR, Hammock BD. 1-Aryl-3-(1-acylpiperidin-4-yl) urea inhibitors of human and murine soluble epoxide hydrolase: structure–activity relationships, pharmacokinetics, and reduction of inflammatory pain. J Med Chem. 2010;53(19):7067–7075. 20812725 10.1021/jm100691c PMC3285450

[41] Perez F, Hujer AM, Hujer KM, Decker BK, Rather PN, Bonomo RA. Global challenge of multidrug-resistant *Acinetobacter baumannii*. Antimicrob Agents Chemother. 2007;51(10):3471–3484. 17646423 10.1128/AAC.01464-06 PMC2043292

[42] Hidron AI, Edwards JR, Patel J, Horan TC, Sievert DM, Pollock DA, Fridkin SK, Participating National Healthcare Safety Network Facilities. NHSN annual update: antimicrobial-resistant pathogens associated with healthcare-associated infections: annual summary of data reported to the National Healthcare Safety Network at the Centers for Disease Control and Prevention, 2006–2007. Infect Control Hosp Epidemiol. 2008;29(11):996–1011. 18947320 10.1086/591861

[43] Prestinaci F, Pezzotti P, Pantosti A. Antimicrobial resistance: a global multifaceted phenomenon. Pathog Glob Health. 2015;109(7):309–318. 26343252 10.1179/2047773215Y.0000000030 PMC4768623

[44] Rauf MK, Talib A, Badshah A, Zaib S, Shoaib K, Shahid M, Flörke U, Imtiaz-Ud-Din Iqbal J. Solution-phase microwave assisted parallel synthesis of N,N′-disubstituted thioureas derived from benzoic acid: biological evaluation and molecular docking studies. Eur J Med Chem. 2013;70:487–496. 24185379 10.1016/j.ejmech.2013.10.012

[45] Obradović D, Nikolić S, Milenković I, Milenković M, Jovanović P, Savić V, Roller A, Đorđić Crnogorac M, Stanojković T, Grgurić-Šipka S, et al. Synthesis, characterization, antimicrobial and cytotoxic activity of novel half-sandwich Ru (II) arene complexes with benzoylthiourea derivatives. J Inorg Biochem. 2020;210:111164. 32634653 10.1016/j.jinorgbio.2020.111164

[46] Encinas M, Iglesias M, Liu Y, Wang H, Muhaisen A, Ceña V, Gallego C, Comella JX. Sequential treatment of SH-SY5Y cells with retinoic acid and brain-derived neurotrophic factor gives rise to fully differentiated, neurotrophic factor-dependent, human neuron-like cells. J Neurochem. 2000;75(3):991–1003. 10936180 10.1046/j.1471-4159.2000.0750991.x

[47] Masters JR. HeLa cells 50 years on: the good, the bad and the ugly. Nat Rev Cancer. 2002;2(4):315–319. 12001993 10.1038/nrc775

[48] Reers M, Smith TW, Chen LB. J-aggregate formation of a carbocyanine as a quantitative fluorescent indicator of membrane potential. Biochemistry. 1991;30(18):4480–4486. 2021638 10.1021/bi00232a015

[49] Abdelhaleem EF, Abdelhameid MK, Kassab AE, Kandeel MM. Design and synthesis of thienopyrimidine urea derivatives with potential cytotoxic and pro-apoptotic activity against breast cancer cell line MCF-7. Eur J Med Chem. 2018;143:1807–1825. 29133058 10.1016/j.ejmech.2017.10.075

[50] Esteves-Souza A, Pissinate K, Graça Nascimento MD, Grynberg NF, Echevarria A. Synthesis, cytotoxicity, and DNA-topoisomerase inhibitory activity of new asymmetric ureas and thioureas. Bioorg Med Chem. 2006;14(2):492–499. 16183295 10.1016/j.bmc.2005.08.031

[51] Charifson PS, Grillot A-L, Grossman TH, Parsons JD, Badia M, Bellon S, Deininger DD, Drumm JE, Gross CH, LeTiran A, et al. Novel dual-targeting benzimidazole urea inhibitors of DNA gyrase and topoisomerase IV possessing potent antibacterial activity: intelligent design and evolution through the judicious use of structure-guided design and structure–activity relationships. J Med Chem. 2008;51(17):5243–5263. 18690678 10.1021/jm800318d

[52] Alfonso EE, Deng Z, Boaretto D, Hood BL, Vasile S, Smith LH, Chambers JW, Chapagain P, Leng F. Novel and structurally diversified bacterial DNA gyrase inhibitors discovered through a fluorescence-based high-throughput screening assay. ACS Pharmacol Transl Sci. 2022;5(10):932–944. 36268121 10.1021/acsptsci.2c00113 PMC9578135

[53] Kundu D, Zhu A, Kim E, Paudel S, Jang C-G, Lee YS, Kim K-M. Potential functional role of phenethylamine derivatives in inhibiting dopamine reuptake: structure–activity relationship. Biomol Ther. 2023;31(1):108–115. 10.4062/biomolther.2022.047 PMC9810443 36098044

[54] Khan T, Sankhe K. DNA gyrase inhibitors. In: Offermanns, editor. Encyclopedia of molecular pharmacology. Cham: Springer International Publishing; 2021. p. 1–8.

[55] Dighe SN, Collet TA. Recent advances in DNA gyrase-targeted antimicrobial agents. Eur J Med Chem. 2020;199:112326. 32460040 10.1016/j.ejmech.2020.112326

[56] Morgan H, Lipka-Lloyd M, Warren AJ, Hughes N, Holmes J, Burton NP, Mahenthiralingam E, Bax BD. A 2.8 Å structure of zoliflodacin in a DNA cleavage complex with *Staphylococcus aureus* DNA gyrase. Int J Mol Sci. 2023;24(2):1634. 36675148 10.3390/ijms24021634 PMC9865888

[57] Shyian M, Shore D. Approaching protein barriers: emerging mechanisms of replication pausing in eukaryotes. Front Cell Dev Biol. 2021;9:672510. 34124054 10.3389/fcell.2021.672510 PMC8194067

[58] Nitiss JL. Targeting DNA topoisomerase II in cancer chemotherapy. Nat Rev Cancer. 2009;9(5):338–350. 19377506 10.1038/nrc2607 PMC2748742

[59] Francisco GD, Li Z, Albright JD, Eudy NH, Katz AH, Petersen PJ, Labthavikul P, Singh G, Yang Y, Rasmussen BA, et al. Phenyl thiazolyl urea and carbamate derivatives as new inhibitors of bacterial cell-wall biosynthesis. Bioorg Med Chem Lett. 2004;14(1):235–238. 14684334 10.1016/j.bmcl.2003.09.082

[60] Webster J, Piscitelli G, Polli A, Ferrari CI, Ismail I, Scanlon MF. A comparison of cabergoline and bromocriptine in the treatment of hyperprolactinemic amenorrhea. N Engl J Med. 1994;331(14):904–909. 7915824 10.1056/NEJM199410063311403

[61] Molinoff PB, Axelrod J. Biochemistry of catecholamines. Annu Rev Biochem. 1971;40(1):465–500. 4399447 10.1146/annurev.bi.40.070171.002341

